# GRAbB: Selective Assembly of Genomic Regions, a New Niche for Genomic Research

**DOI:** 10.1371/journal.pcbi.1004753

**Published:** 2016-06-16

**Authors:** Balázs Brankovics, Hao Zhang, Anne D. van Diepeningen, Theo A. J. van der Lee, Cees Waalwijk, G. Sybren de Hoog

**Affiliations:** 1 CBS-KNAW Fungal Biodiversity Centre, Utrecht, the Netherlands; 2 Institute of Biodiversity and Ecosystem Dynamics, University of Amsterdam, Amsterdam, the Netherlands; 3 State Key Laboratory for Biology of Plant Diseases and Insect Pests, Institute of Plant Protection, Chinese Academy of Agriculture Sciences, Beijing, China; 4 Wageningen University and Research Centre, Wageningen, the Netherlands; University of Canterbury, NEW ZEALAND

## Abstract

GRAbB (Genomic Region Assembly by Baiting) is a new program that is dedicated to assemble specific genomic regions from NGS data. This approach is especially useful when dealing with multi copy regions, such as mitochondrial genome and the rDNA repeat region, parts of the genome that are often neglected or poorly assembled, although they contain interesting information from phylogenetic or epidemiologic perspectives, but also single copy regions can be assembled. The program is capable of targeting multiple regions within a single run. Furthermore, GRAbB can be used to extract specific loci from NGS data, based on homology, like sequences that are used for barcoding. To make the assembly specific, a known part of the region, such as the sequence of a PCR amplicon or a homologous sequence from a related species must be specified. By assembling only the region of interest, the assembly process is computationally much less demanding and may lead to assemblies of better quality. In this study the different applications and functionalities of the program are demonstrated such as: exhaustive assembly (rDNA region and mitochondrial genome), extracting homologous regions or genes (IGS, RPB1, RPB2 and TEF1a), as well as extracting multiple regions within a single run. The program is also compared with MITObim, which is meant for the exhaustive assembly of a single target based on a similar query sequence. GRAbB is shown to be more efficient than MITObim in terms of speed, memory and disk usage. The other functionalities (handling multiple targets simultaneously and extracting homologous regions) of the new program are not matched by other programs. The program is available with explanatory documentation at https://github.com/b-brankovics/grabb. GRAbB has been tested on Ubuntu (12.04 and 14.04), Fedora (23), CentOS (7.1.1503) and Mac OS X (10.7). Furthermore, GRAbB is available as a docker repository: brankovics/grabb (https://hub.docker.com/r/brankovics/grabb/).

This is a *PLOS Computational Biology* Software paper

## Introduction

High throughput sequencing of whole genomes is becoming routine due to the advances in sequencing technologies and the reduction of the costs involved. Currently, the number of genome and transcriptome sequencing datasets made accessible for the research community is steadily increasing [1].

However, in many studies using NGS data, the main focus is on the nuclear genome. This can be seen on the relatively low number of organellar genome assemblies published compared to the number of nuclear genome assemblies published. The mitogenome (mitochondrial genome) and the ribosomal DNA repeat region (18S rRNA—ITS1—5.8S rRNA—ITS2—28S rRNA— IGS) are generally not completely assembled, even when there is sufficient information in the NGS data [2]. These regions contain loci that are predominantly used for phylogenetic comparisons and species identification. Furthermore, there is no program that promises to extract barcoding loci form NGS reads.

The time and computational power required for assembling a specific region of the genome is less than that required for assembling the entire genome, while the quality of the assembled sequences may improve. Few resources exist to examine regions other than the nuclear genome. So far there is only one program, MITObim, which selectively assembles organellar genomes, and was developed for the mitogenome [2]. While other programs are aimed at re-assembly to improve an existing assembly [3] or at closing gaps [4], MITObim is designed to use the sequence of a related species as a reference for the assembly until either one of two completion criteria is met: i) a maximum number of iterations specified at invocation is reached, or ii) no new reads are found.

Here, we present GRAbB (Genomic Region Assembly by Baiting), a new versatile program that is designed to be flexible in terms of input options, assembly and completion criteria. The program uses a reference file to identify reads corresponding to the target region. For the assembly, the program can use two well-known assemblers, Edena [5, 6] and Velvet [7], but the source code is designed to be easily expandable to employ other assemblers, as demonstrated in the documentation. It is the first program that can handle multiple regions separately within a single run. Moreover, it is the first program that is able to extract homologous sequence regions, such as barcoding sites.

In this paper, the exhaustive assembly capabilities of GRAbB were compared with that of MITObim. Besides this comparison, GRAbB was also run on NGS data to demonstrate its functionalities targeting multiple regions with diverse completion criteria.

## Design and Implementation

### Algorithm overview

GRAbB is written in Perl and it uses only modules that are part of the core distribution of Perl. In addition to basic UNIX commands, the following third-party programs are used by GRAbB: mirabait (from the MIRA package; [8]), Seqtk (https://github.com/lh3/seqtk), Edena [5, 6], Velvet [7] and Exonerate [9].

The program is designed to be versatile and flexible with the following functionalities:

- To use pairing information,
- To use additional bait sequences,
- To assemble multiple regions separately in a single run,
- To use any of a range of completion criteria (different ones for each region are allowed in a multi-assembly run).

These functionalities are detailed below at the appropriate steps of the algorithm.

### Input

**Fig 1:**
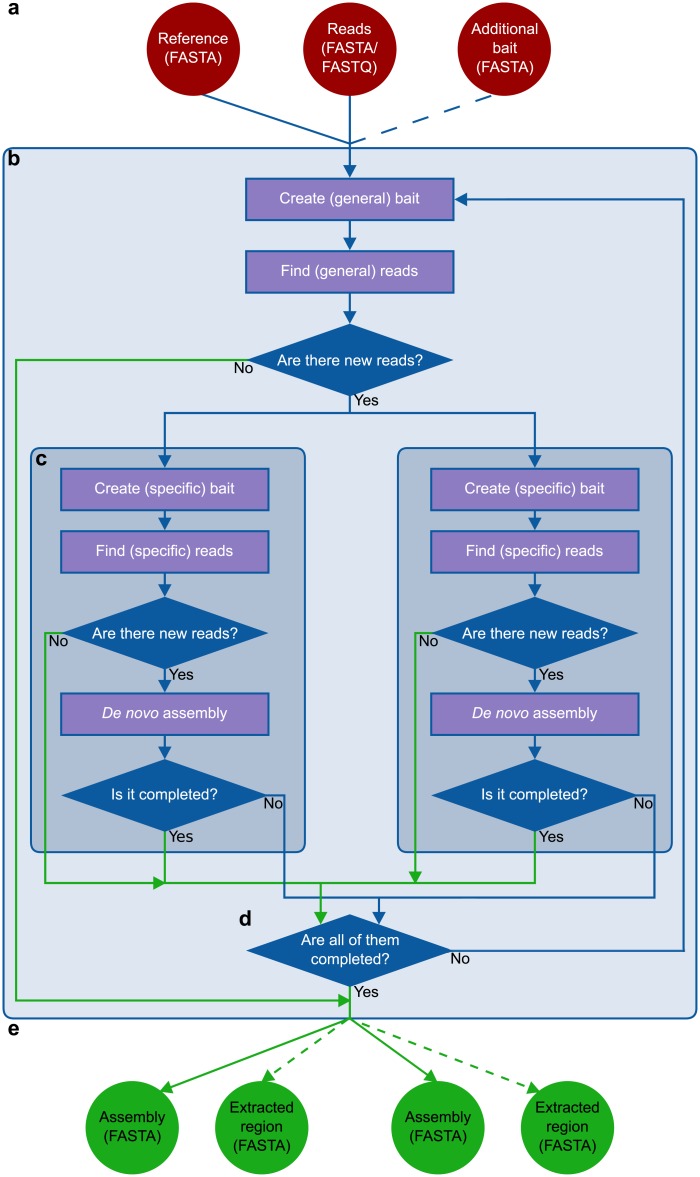
Schematic workflow of GRAbB. **a:** The input files for GRAbB. **b:** The main loop of GRAbB. **c:** Internal loops for the individual threads, the number of threads is based on the number of sequences in the reference file in multi-mode, otherwise there is only a single thread. **d:** Check whether there are incomplete threads left or not. **e:** Output files for each thread. Dashed arrows indicate optional files (only in exonerate-mode). Green arrows indicate termination signal of a given thread or run. If GRAbB is not run in multi-mode, then the first step within the internal loop is the *de novo* assembly, the preceding ones are skipped.

As shown in Fig 1a, there are two mandatory input files (the reference file and at least one read file), In addition, there is an optional input file (the additional bait file).

The reference file may contain multiple sequence entries, which serve as references for the individual threads if the program is run in multi-mode. The description lines may contain specification for the completion criterion to be used for the given sequence, allowing users to use different completion criteria for the different threads within a single run.

Multiple read files can be specified as input. GRAbB can make use of pairing information.

The additional bait file is used for the first baiting step by merging it with the reference to create a unified bait file. The file may contain more than one sequence.

### Main loop

The main loop of GRAbB can be summarized as follows: i) creating the bait file, ii) finding reads by baiting, iii) *de novo* assembly of the selected reads, and iv) testing completion (Fig 1b). When multi-mode is selected, the general baiting step is followed by specific baiting, *de novo* assembly and completion testing steps for each of the threads (Fig 1c). The threads are generated by splitting the reference file into single-entry FASTA files. These newly created reference files are used as bait for the initial specific baiting steps for the given thread (Fig 1c). At the end of each cycle of the main loop, the program checks whether there is any thread that is not completed yet: then it continues or stops accordingly (Fig 1d).

#### i) Creating the bait file

Before the first baiting step, the specified reference and the additional bait file are combined into a single internal bait file. In multi-mode, the reference sequences corresponding to the given thread will be used for the first specific baiting step.

In each of the later iterations of the program, the latest assembly is used as (internal) bait file. For the general baiting step in multi-mode the individual assembly files are combined into a single (general) bait file.

#### ii) Finding reads

Reads corresponding to the target sequence are identified by using exact k-mer (31 bp) matching that is implemented by mirabait. The names of the reads thus identified are collected and added to the list of read names from previous iterations (there is a separate list for general baiting and for each of the threads.) If there are no new reads identified, the program stops the iteration: either for the given thread or for all the threads, depending on which baiting step generated this result. The identified reads are collected from the read files using Seqtk into internal read files.

By using a general baiting step before the specific baiting, it is possible to reduce the required runtime, since the large input read file(s) is/are only screened once per iteration and the specific baiting is confined to screening the reads that are already identified to be specific during the general baiting.

#### iii) *De novo* assembly

The program can use two assemblers, Edena and Velvet, by default, however there is a skeleton code to add other assemblers to be used by the program. Even though Edena is the default assembler, by using command line options other assemblers may be selected as well.

The fact that single- or paired-mode is selected is passed on to the assembler program that assembles the specific reads *de novo*. Furthermore, additional arguments may be passed, such as overlap cutoff size (Edena), to the assembler program at the invocation of the main program.

At the invocation of GRAbB it is possible to specify a length filter that excludes the contigs from the assembly that are shorter than the specified length from the completion testing, and these are not used for generating the bait file for the next iteration either. This enables to minimize the effect of repeat regions on the assembly process.

#### iv) Testing completion

There are multiple completion criteria that can be specified for the program. These can be specified for each thread separately or for all of the threads at once. In addition, it is possible to specify multiple criteria for the same thread or run. The program stops when any one of these criteria is met.

The first completion criterion is implicit: the program stops if no new information is found. This means that either there are no new reads found or that the new assembly is identical to the bait used for the current iteration. If no other criterion is specified, then the run will be exhaustive, since it iterates until it cannot find any new information.

The simplest explicit completion criterion is the size criterion. There are three options that can be used for this setting: i) total assembly size, ii) length of the longest contig, and iii) N50 value of the assembly. This criterion is tested independently for each of the threads in multi-mode, or alternatively for the single thread. Multiple criteria can be used in a single run; this also applies to the different size criteria. These settings are useful when exploring the vicinity of a specified sequence region.

The final criterion is matching a homologous sequence. In this case, the specific reference sequence is used to identify the homologous region within the assembly. In order to identify the matching region, GRAbB uses Exonerate with settings that ensure that the entire reference sequence is aligned to the contigs. This makes it possible to match sequences that are somewhat dissimilar to the reference and may contain indels, causing gaps in the alignment. If the reference could be aligned to the contigs over its entire length, the completion criterion is met, and the matched region is extracted from the assembly in the same orientation as the reference sequence and saved to a separate output file. Otherwise, the length of the matched region is compared to that of the previous iteration. In case the length of the matched region has increased, the thread or run will continue, otherwise the thread or run will stop.

### Up- and downstream processes

Besides the main program, there are some helper programs included within the package that can help with preprocessing the input data or to further process the output data. These are detailed in the documentation (https://github.com/b-brankovics/grabb).

## Results

### Simulated data

To compare the performance of GRAbB with that of MITObim 1.7 (using MIRA 4.0.2; https://github.com/chrishah/MITObim.git; [2]), simulated sequencing data generated based on the *F. graminearum* mitogenome sequence available from NCBI (NC_009493) were used together with either the original *F. graminearum* (NC_009493) or *F. oxysporum* (NC_017930) mitogenome as reference. MITObim has two assembly settings: *de novo* and mapping. However, since the mapping setting did not prove to be applicable to reconstruct the *F. graminearum* sequence by using *F. oxysporum* as reference, only the *de novo* setting was used for the comparison. GRAbB produced a single contig in both cases. MITObim produced multiple contigs in both runs, however a single contig could be obtained from the LargeContigs MAF format file produced by using miraconvert. All the single contigs produced could be circularized using the merge_contigs.pl and were identical to the original sequence. Although both MITObim and GRAbB could assemble the genome correctly, GRAbB did it more efficiently and demanded less disk space for the output folders Table 1 (sequence similarity information is in supplementary file S1 Text).

**Table 1:** Comparison between GRAbB and MITObim using our *in silico* generated paired-end read library.

Program	Reference	N	CPU usage (%)*	Time elapsed (m:s)	Maximum memory usage (Mb)	Output folder size (Mb)
GRAbB	*F. graminearum*	2	157	00:15.52	285.09	70
GRAbB	*F. oxysporum*	10	161	01:49.24	286.05	527
MITObim	*F. graminearum*	3	150	09:42.15	1,354.86	4,126
MITObim	*F. oxysporum*	9	150	25:37.71	1,358.81	10,667

Data were generated by /usr/bin/time on Ubuntu 14.04.1. Both programs were run on the same simulated read library derived from *F. graminearum* mitogenome. Reference column shows which mitogenome was the input for the run. N is the number of iteration it took to complete the assembly. Output folder size refers to the disk space occupied by the output files of the run. This was assessed using /usr/bin/du on Ubuntu 14.04.1.

* The program, /usr/bin/time, assigns 100% for each of the cores of the processor, and the computer used has four cores, thus the maximal CPU usage is 400%.

### NGS data

To demonstrate the capabilities of GRAbB, it was run on paired-end reads derived from *Fusarium oxysporum* f. sp. *cubense* race 4 strain B2 (SRR550152; [10]) and the results were compared to the assembly published for this strain (AMGQ01). In the single run multiple regions were targeted simultaneously: mitochondrial genome, rDNA region, barcoding loci (IGS, TEF1a, RPB1 and RPB2). The goal of the threads were as follows: assemble the complete mitogenome, assemble the complete rDNA region, extract barcoding loci, compare the extraction results using sequences from different species and test the effect on the extraction when the query contains a gap (Table 2; sequence similarity information in supplementary file S1 Text).

**Table 2:** Overview of the multi-query run.

Thread number	Target	Reference				Result size (bp)	Assembly type	Number of iterations	
		Region	Species	Accession number	Size (bp)			Specific	General
1	mt	mt	*F. oxysporum*	NC_017930	34,477	49,697	exhaustive	18	3
2	rDNA	IGS	*F. oxysporum*	FD_00403_IGS	1,449	7,872	exhaustive	2	10
3	IGS	IGS	*F. oxysporum*	FD_00403_IGS	1,449	1,446	exonerate	1	1
4	TEF1a	TEF1a	*F. oxysporum*	FD_00403_EF-1a	689	684	exonerate	1	1
5	TEF1a	TEF1a	*F. oxysporum*	FD_00403_EF-1a*	652	647	exonerate	1	1
6	TEF1a	TEF1a	*F. graminearum*	FD_00001_EF-1a	643	647	exonerate	3	1
7	TEF1a	TEF1a	*F. solani*	FD_01036_EF-1a	677	647	exonerate	4	1
8	RPB1	RPB1	*F. oxysporum*	FD_02003_RPB1	1,606	1,606	exonerate	1	1
9	RPB2	RPB2	*F. oxysporum*	FD_02003_RPB2	1,762	1,899	exonerate	1	1
10	RPB2	RPB2	*F. oxysporum*	FD_00120_RPB2-57 FD_00120_RPB2-711	879 860	1,876	exonerate	1	1

The accession numbers are either GenBank accessions or FusariumID (http://isolate.fusariumdb.org) accessions. The assembly type specifies which assembly mode was selected for the given thread during the GRAbB run. Multiple specific iterations are run only during the first general iteration.

*: This sequence was truncated to span the same region as FD_00001_EF-1a and FD_01036_EF-1a.

#### Mitochondrial genome

The mitochondrial genome of the strain was assembled into a single contig, which had 9-9 oligoC sequences at both ends. To solve this problem, Edena was rerun on the sequences specific for the mitogenome, but the reads were trimmed to be 70 bp long. By removing part of the ‘3 end of the reads, the assembly improved. The final assembly contained a single contig, and the two ends of the contig overlapped by 60 bp, indicating a circular nature. This is in agreement with the fact that the mitochondrial genome is circular in *Fusarium* spp [11].

The circularized sequence was 49697 bp long (LT571433). The reference sequence (NC_017930) lacked a region (approximately 12 kbp long) that is present in the mitogenomes of all other *Fusarium* spp. sequenced thus far [11]. This region contains tRNA genes and a large ORF (open reading frame) that is typical for *Fusarium* species, the function of this ORF is unknown. The new sequence contained this region, in addition to three new introns in protein coding genes.

In the AMGQ01 assembly there are 30 contigs containing mitochondrial sequences. These sequences cover only 39.07% of the mitogenome.

#### Ribosomal DNA region (18S rRNA—ITS1—5.8S rRNA—ITS2—28S rRNA—IGS)

The assembly for the rDNA region contained two contigs, and the contigs could be joined because they overlapped by 99 bp, indicating a circular or repetitive morphology. The ribosomal DNA region is not circular, but since it is present within the genome in multiple copies as direct repeats [12] it can be considered as a circular sequence from a bioinformatics perspective.

The circularized sequence was 7,872 bp long. The obtained sequence (LT571434) contained the 18S, 5.8S and 28S rRNA genes, as well as, the ITS1, ITS2 and IGS regions. Since the rDNA region is present in multiple copies in the nuclear genome, therefore has higher coverage compared to surrounding regions. This makes it possible to assemble the complete repeat region without assembling the flanking regions. In the AMGQ01 assembly there are 14 contigs containing rDNA region sequences. These sequences cover only 63.89% of the rDNA region.

#### Barcoding loci (IGS, TEF1a, RPB1 and RPB2)

All the threads targeting barcoding loci finished by matching the query sequence in the assemblies. The result for the threads of the three TEF1a sequences from different species (*F. oxysporum*, *F. graminearum* and *F. solani*) returned identical sequences. The IGS sequence had only partial matches when compared with the AMGQ01 assembly, but matched completely with the newly assembled rDNA region sequence. The TEF1a, RPB1 and RPB2 sequences were identical to matching sequences from the assembly (AMGQ01), however, the RPB2 locus was found at the junction of two contigs in the assembly, which overlapped by 5 bp.

### Conclusions

GRAbB (Genomic Region Assembly by Baiting) is a new versatile program that is designed to be flexible in terms of input options, assembly and completion criteria. It is the first program that can handle multiple regions separately within a single run. Moreover, it is the first program that is able to extract homologous sequence regions, such as barcoding sites.

The program was compared with MITObim, which is meant for the exhaustive assembly of a single target based on a similar query sequence. GRAbB is shown to be more efficient than MITObim in terms of speed, memory and disk usage.

The other functionalities, handling multiple targets simultaneously and extracting homologous regions, of the new program are not matched by other programs. These functionalities were demonstrated by a single run. GRAbB managed to assemble the individual target regions independently of each other. In the case of the mitochondrial genome, the rDNA region and last RPB2 example, the complete sequences could be recovered, although the target sequences contained regions that were not present in the query sequences. The same TEF1a region was targeted by three homologous query sequences from three different species, although the sequences shared different levels of similarity to the target sequence, all three threads returned the same sequence. GRAbB identifies reads corresponding to a target region by using exact 31-mer matching, thus 31-basepair-long sequence identity between query and target can be sufficient to seed an assembly.

## Availability and Future Directions

GRAbB is available with explanatory documentation at https://github.com/b-brankovics/grabb. Furthermore, GRAbB is available as a docker repository: brankovics/grabb (https://hub.docker.com/r/brankovics/grabb/). The GitHub package has been tested on Ubuntu (12.04 and 14.04), Fedora (23), CentOS (7.1.1503) and Mac OS X (10.7). The docker repository can be used on any platform that has a working installation of docker. The program is made available with MIT license.

GRAbB is written in a way that allows users to run it with any assembler that can be executed via the command line. Although, the program is meant to be run with *de novo* assemblers, the skeleton code provided in the package and convention of creation and location of intermediary files allow users to employ reference based assemblers, as well.

## Supporting Information

S1 Text Sequence similarity between target and query sequences. (PDF)
