# Supplementary material for: GRAbB: Selective Assembly of Genomic Regions, a New Niche for Genomic Research
Source: PLoS Comput Biol. 2016 Jun 16;12(6):e1004753. doi: 10.1371/journal.pcbi.1004753 (PMC4911045; doi:10.1371/journal.pcbi.1004753)
Supplement: S1 Text — (PDF) [file pcbi.1004753.s001.pdf]

Sequence similarity between target and query sequences

Sequence similarity between query and target have been calculate by two approaches: sequence alignment and 31-mer comparison.

Sequence alignment: Target and query have been aligned using MUSCLE [1,2] or FSA [3], and the identical positions have been reported. The number of identical positions have been divided by the length of the alignment to calculate the percentage.

31-mer approach: The number of 31-mers shared by the query and target have been calculated and reported. The percentage is calculate by dividing the number of shared 31-mers with the number of unique 31-mers in the target sequence.

Table 1: Sequence similarity of published mitochondrial genomes

**Species**	**Accession number**	**Length (bp)**	**Identical (bp)**	**Identical (%)**	**Shared 31-mers**	**31-mer identity (%)**
F. graminearum	NC_009493	95676	95676	100.00	189584	100.00
F. oxysporum	NC_017930	47409	24669	24.04	18304	9.65

The accession numbers are GenBank accessions.

Table 2: Sequence similarity of TEF1a sequences

**Species**	**Accession number**	**Length (bp)**	**Identical (bp)**	**Identical (%)**	**Shared 31-mers**	**31-mer identity (%)**
F. oxysporum	AMGQ01*	647	647	100.00	1234	100.00
F. oxysporum	FD_00403_EF-1a*	652	636	97.40	702	56.89
F. graminearum	FD_00001_EF-1a	643	524	77.86	200	16.21
F. solani	FD_01036_EF-1a	677	499	71.49	232	18.87

The accession numbers are either GenBank accessions or FusariumID (http://isolate.fusariumdb.org) accessions.
*: This sequence was truncated to span the same region as FD_00001_EF-1a and FD_01036_EF-1a.

Table 3: Sequence similarity between queries and targets in the multi-query run.

**Thread number**	**Target**	**Species**	**Accession number**	**Query size (bp)**	**Target size (bp)**	**Identical (bp)**	**Identical (%)**	**Shared 31-mers**	**31-mer identity (%)**
1	mt	*F. oxysporum*	NC_017930	34,477	49,697	24669	65.24	45522	46.17
2	rDNA	*F. oxysporum*	FD_00403_IGS	1,449	7,872	1406	17.85	1336	8.49
3	IGS	*F. oxysporum*	FD_00403_IGS	1,449	1,446	1406	96.90	1336	47.18
4	TEF1a	*F. oxysporum*	FD_00403_EF-1a	689	684	673	97.54	774	59.17
5	TEF1a	*F. oxysporum*	FD_00403_EF-1a*	652	647	636	97.40	702	56.89
6	TEF1a	*F. graminearum*	FD_00001_EF-1a	643	647	524	77.86	200	16.21
7	TEF1a	*F. solani*	FD_01036_EF-1a	677	647	499	71.49	232	18.80
8	RPB1	*F. oxysporum*	FD_02003_RPB1	1,606	1,606	1590	99.00	2090	66.31
9	RPB2	*F. oxysporum*	FD_02003_RPB2	1,762	1,899	1753	92.31	2902	77.64
10	RPB2	*F. oxysporum*	FD_00120_RPB2-57<br>FD_00120_RPB2-711	879<br>860	1,876	1724	91.90	2486	67.33

The accession numbers are either GenBank accessions or FusariumID (http://isolate.fusariumdb.org) accessions.
*: This sequence was truncated to span the same region as FD_00001_EF-1a and FD_01036_EF-1a.

References

[1] Edgar RC. MUSCLE: multiple sequence alignment with high accuracy and high throughput. Nucleic Acids Res. 2004 Mar;32(5):1792–7.

[2] Edgar RC. MUSCLE: a multiple sequence alignment method with reduced time and space complexity. BMC Bioinformatics. 2004 Aug;(5):113.

[3] Bradley RK, Roberts A, Smoot M, Juvekar S, Do J, Dewey C, et al. Fast statistical alignment. PLoS Comput Biol. 2009 May;5(5):e1000392.
